# A Qualitative Study on the Pathways to Evidence-Based Antenatal Care in Periurban Ghana

**DOI:** 10.1155/2018/4381708

**Published:** 2018-07-12

**Authors:** Jones Asafo Akowuah, Peter Agyei-Baffour, Benedict Osei Asibey

**Affiliations:** ^1^Department of Agricultural Economics, Agribusiness and Extension, Kwame Nkrumah University of Science and Technology, Kumasi, Ghana; ^2^Department of Community Health, Kwame Nkrumah University of Science and Technology, Kumasi, Ghana; ^3^Department of Geography and Rural Development, Kwame Nkrumah University of Science and Technology, Kumasi, Ghana

## Abstract

Maternal health care has become a major concern on international fora in the 21st century. Even though major interventions have been taken to scale up maternal health care locally, nationally, and globally, adequate utilisation has not been achieved due to system-induced setbacks, especially in sub-Saharan Africa. The study explored the facilitators and barriers to antenatal care use in periurban Ghana. Seventeen (17) respondents consisting of four mothers receiving ANC services, four mothers receiving postnatal care with their ANC experience, four midwives, and four nurses with the District Public Health Nurse were involved in the study. The exploratory case study design was used with respondents comprising two focus groups and interview participants. Using thematic analysis, the results revealed that restrictive factors like travel time, long waiting time, transport cost, service cost, quality of service, and attitude of hospital staff still act as constraining factors even after the introduction of free maternal health care. The study concludes that practices like focused ANC and routine monitoring to facilities among others have increased utilisation. The study therefore recommends that to ensure adequate utilisation, the government and other stakeholders should offer support to the less-privileged mothers. Again, services should be easily available at facilities to pregnant women even if they are to be bought. It is further recommended that antenatal care services should be reoriented and clinical psychologists should be placed at all health centres to empower health staff on the best attitude towards clients. Interventions if mainstreamed into the national maternal health policy could be useful.

## 1. Background

Good health is a key human development indicator, and it ensures quality of life and the capacity to participate in productive activities [1]. Good health ensures people's involvement in all sectors of economy and increased well-being through wealth creation. Good health of women will also act as an important component in the measurement of human capital [2]. Therefore, all means to ensure good health, which includes the provision and use of health-care services, including maternal health services thus emerges as an important public health and policy issue and a matter of concern to stakeholders. This reflects in efforts to improve health outcomes and to meet international obligations to make health care broadly accessible [3, 4].

Improved and effective antenatal care brings desired outcomes to pregnant women and their babies [5]. A good start in life means empowering women and children as health impression of early development and education of people last in generations [6, 7]. According to Mason et al. [8], about 80% of maternal deaths occur worldwide through pregnancy complications, delivery, and the period of postpartum. It is widely observed in developing countries that structural, personal, and service-related factors influence the rate antenatal care is accessed and used [9]. However, these acknowledged factors can be addressed by considering the realities on the ground in local settings.

Maternal mortality remains a dilemma especially in the sub-Saharan Africa in spite of efforts geared at improving maternal health [8]. It is estimated that over 500,000 women die each year out of pregnancy-related complications worldwide [10–12]. Most of these deaths occur in the sub-Saharan Africa [13]. According to the Reproductive and Child Health unit of the Ghana Health Service [14], maternal mortality is estimated 230 per 100,000 live births.

However, these complications could be solved totally or reduced to the barest minimum if there are skilled health personnel with the best work ethics operating in the finest of environments [13, 15, 16]. There is therefore the call to various governments to strengthen the upstream approach to maternal health-care services. This call will increase, empower, and sustain the capabilities of skilled birth attendants (doctors, nurses, and midwives). This will then have spillover effects on women to gaining control over their health through health sensitisation given to them by these skilled birth attendants [17]. Hence, the well-being of the life of women depends on their health and education [18].

Various qualitative studies conducted in most developing countries indicate both formal and informal structures to maternal care either facilitate or impede health care [19, 20]. It is therefore necessary and proper to combine both local and scientific expertise to addressing the lapses in utilising maternal health. This could be an upstream approach to understanding the realities in maternal health and resorting to the best practices in maternal care use. This would increase maternal health-care use to achieve the WHO recommendations, which peg its ANC minimum requirement of four visits of which the pregnant mother is believed to have fully utilised the ANC.

According to the Ghana Ministry of Health [21], the country now has a 3-tier health-care system; primary health care is the basic unit, which is the “first” level of contact between the individual and the health system where majority of prevailing health problems can be satisfactorily managed and the closest to the people. The next is the secondary health care, which is the 1st referral level, provided by the district hospitals where more complex problems are dealt with and comprises curative services. The last phase is the tertiary health care which offers superspecialist care. The tertiary health-care services are provided by the regional and central level institution and provide training programmes.

In Ghana, the free maternal health-care policy was introduced in September 2003 as a pilot project in the central, upper east, upper west, and the northern regions. It was later scaled up to the six remaining regions in April 2005. All services except laboratory tests for pregnant mothers are free. Among other benefits of the policy is the National Health Insurance Scheme (NHIS) which offers low-risk pregnancy care undertaken by midwives and high-risk pregnancy care by consultants and other skilled health personnel [21]. According to the Multiple Indicator Cluster Survey conducted in Ghana by the Ghana Statistical Service [22], the rate of ANC use is 87 percent. Ideally, ANC services in Ghana are free; but in reality, all pregnant women must register with the NHIS for paying only the cost of transaction fee (around USD 1). Also, facilities require some payments for the recommended laboratory tests and screenings from pregnant women. In addition, the Ghana MoH approves free delivery services as components of the free maternal health-care policy. However, women are usually asked to pay a fee (around USD 10) when referrals are arranged on caesarean sections, among other things to cover the delivery supplies of cotton wool and gloves. All these payments burden pregnant women and mostly deter or limit them in their use of ANC services, let alone utilising the services to the recommended visits by the WHO.

Extensive quantitative studies have been carried out to determine why most pregnant women fail to utilise the four ANC visits that the WHO recommends. A recent study conducted by Gabrysch and Campbell [23] in low- and middle-income countries revealed a “significant unexplained community level variation” to explain maternal health-care delivery which could be explained by “measurement or omission error.” This anomaly explains the need to consider local contexts and the dynamics they bring to the fore. These issues need to be explored using qualitative methods. This study seeks to explore the factors that either facilitate or impede antenatal care use in the context of Kwabre East District of Ghana.

## 2. Methods

Using qualitative study design, this study sought to explore the factors that either empower or deter women with diverse living conditions in the Kwabre East District to utilise the free maternal health services offered for pregnant women under the NHIS. It presents findings from 2 focus groups with 8 participants each and 1 interview schedule carried out in the Kwabre East district of Ghana.

### 2.1. Study Setting

**Figure 1:**
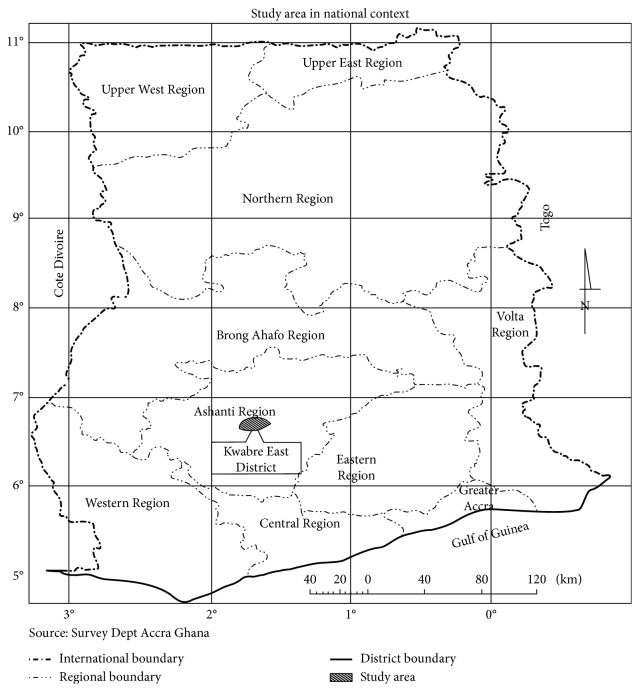
Study district in national context. Source: Kwabre East District Assembly [24].

The study was conducted in the Kwabre East District. The district is among the twenty-seven administrative districts in Ashanti Region and has a total land area of 123 square kilometres constituting approximately 0.51 percent of the total land area of the Ashanti Region (24,370.5 square kilometres). The district lies within latitudes 60°45′ and 60°50′ North and longitudes 10°30′ and 10°35′ West [24]. Even though it mostly has the characteristics of rural districts such as involvement in primary production, wide disparity in wealth, good roads, poor telecommunications, and social services, it is different with regard to its distance from Kumasi, the capital of the Ashanti Region. Its proximity to Kumasi is accompanied with mixed thoughts. On the one hand, it would be denied of many rural development considerations once its status is changed to municipality. On the other hand, it would be able to attract and retain well-trained staff and career officials than most remote districts from Kumasi. Nevertheless, most staff is not pleased to be posted to remote parts of the district. For the purpose of the study, four facilities, namely, Mamponteng, Asonomaso, Antoa, and Sakra Wonoo, all of which are publicly owned facilities, were selected. Asonomaso hospital is the only 2nd tier in the district with the rest rendering primary health services. The district is captured in the national map of Ghana in Figure 1.

### 2.2. Study Procedures

The study recognised mothers who had just delivered and expectant mothers as the study population since they are the main users of maternal health-care services and can hence information-rich population. The study population was, however, sought from the district health information management system software of the Ghana health services (i.e., ghsdhims) [25]. In addition, the study was guided by the principles and strategies adopted by Patton [26].

Qualitative data for the study were collected from the respondents between 15th June and 15th October, 2015. The interview was conducted with the Public District Health Nurse of the District Health Directorate. In addition to this, two focus groups were conducted comprising four expectant mothers and four mothers who had just delivered, and the focus was on their ANC experience. These mothers aged 15–49 were selected through a facility-based maternal care service from the four public facilities that were included in the study. Hence, the sample for the study was purposively taken to comprise the experience of women who received ANC.

To discover the dynamics which stimulate the rate of antenatal care use and the perceptions of mothers in utilising ANC, two focus group discussions were conducted. One was with four pregnant women receiving prenatal care with two nurses and two midwives. The other group had four mothers receiving prenatal care with two nurses and two midwives.

Through the focus groups, women were asked to describe their experiences of pregnancy and antenatal care services received. While motherhood is often associated with a positive and fulfilling experience, for too many women especially in Africa, it is associated with suffering, ill-health, and even death [27–29]. The study therefore sought to unearth these dimensions of maternal health. Hence, the recount of personal experiences began from the first day of conception to the time of interaction, be it still pregnant or delivered. In all, the focus group discussions were conducted in Twi and were later translated and transcribed into English. Since the Twi language used for the study was the mother tongue of the researchers, no interpreters were needed and the resident nurses in the selected facilities acted as facilitators. Study participants were, however, recruited by means of attending ANC services and a seminar organised for the pregnant women and those who had just delivered by the facilities within the district.

To further explore the health-care system provided for maternal mothers, an extensive interview was conducted by the researchers with the District Public Health Nurse. The interview with the Public Health Nurse was conducted in English due to her high proficiency in the language. Hence, interview data were only transcribed afterwards without any ambiguity. The researchers were guided by an interview guide that showed a broad structure of the services and the general health system provided for maternal women in the district. The interview section and focus groups were all audio-recorded and transcribed afterwards. Permission to record the interview was however sought from the participants.

### 2.3. Ethical Consideration

Ethical clearance for this study was sought from the Department of Community Health-KNUST, the Kwabre East District Health Directorate, the Planning Unit of the Kwabre East District Assembly, various selected facility nurses, and health workers in the Kwabre East District. In addition, formal consent was obtained from individual pregnant mothers in the selected health facilities who agreed to be part of the study. Privacy and confidentiality were ensured and maintained. Participants were informed of their right to voluntary participation in the study and to retire at anytime without fear of intimidation.

### 2.4. Analysis

The researchers used thematic analysis to analyse the data obtained. Qualitatively, thematic analysis is the technique which helps to identify, interpret, and report patterns or themes found in one's dataset which portray the experiences, beliefs, and observations of respondents in relation to the epistemological position of one's research question [30]. Thematic analysis is able to offer the systematic element of a research data of one's study and permits the researcher to combine analysis by generating codes with meaning in context and thus adding the advantages of the refinement and complexity of a truly qualitative analysis [31]. Through the emerged patterns of interrelated codes, basic themes and organising themes, we supported our thematic analysis with network analysis which eventually helped us arrive at a global theme.

This study used an inductive theme embedded in thematic analysis of the interpretivism tradition by using the “exact” words used by the participants gathered from the field. Again, the study employed manifest coding in transcription. This technique emphasises the organisation and rich description of the dataset since in qualitative inquiry quotations speak for themselves, which aims to identify themes, stay flexible, and stay open-minded [32]. Hence, the study used thematic analysis as a way of getting closer to the datasets and developing some deeper appreciation of the contents through “thick description.”

We checked all datasets for coherence and consistency to avoid mistakes. Data were then coded by an author and verified by the two authors independently to determine the associated factors which either facilitated or hindered women in utilising ANC services. Most factors appeared to determine the rate of utilisation of antenatal care. After coding was done, the researchers did further checks on the consistency and interpretation of datasets. Hence, transcribed datasets on focus groups were exchanged among researchers for compliance. Finally, codes and analysis of transcribed data were serialised, shared, and discussed [30, 33]. After all focus groups' transcripts had been coded, meaningful codes that emerged from the codes were however grouped into basic themes. Basic themes were also discussed and ordered into organising themes. However, overarching themes were discussed and unanimously agreed upon among authors into a global theme by the researchers. Thematic analysis from the interview set followed similar steps outlined above and were all put together which are outlined in Table 1.

## 3. Findings

In Ghana, the use of antenatal care services is conducted and measured only at the facility level. Thus, the study participants were contacted at the various facilities where ANC was sought where the utilisation levels of respondents were at variance. However, some women used other health-care services in addition to ANC. The table below outlines the details of the study findings.

## 4. Discussion

This paper sought to explore the human- and system-induced factors that act as facilitators or barriers to antenatal care use in the Kwabre East district of Ghana after the introduction of the free maternal health policy. The discussion is involved with women aged 15–49 to whom a publicly funded antenatal care package is offered. The pregnant women with an average age of 26 that were used as study participants were obtained from the district health information management system software of the Ghana health services (i.e., ghsdhims) [25]. This helped to reduce the response bias of the study, and views of respondents were reliably obtained.

### 4.1. Pattern of Antenatal Care Utilisation

In the Kwabre East district, the health directorate provides for varied maternal services including health education to serve the needs and demands of women. It is widely acknowledged that the kind of ANC services provided for women influence their rate of accessing antenatal care during pregnancy [4, 34–38].

*Services! Hmmm; we at the district health directorate offer to our clients multiple of broad services and these include the distribution of health staff, routine monitoring of facilities screening, immunisation, health education and smanagement of minor ailment. Again, errrrr for every fortnight, the health staff in the district embark on health education on zonal basis on errr the services rendered to pregnant mothers and the need to utilise such services.* (District Public Health Nurse)

### 4.2. Socioeconomic Factors Influencing ANC Utilisation

Though many rural and remote residents in the world face considerable challenges in accessing appropriate health services, many of them struggle to secure resources and recruit and retain staff [39]. The health directorate in collaborated efforts with other institutions of state provides services that are captured under the rural health concepts.

*Yes, the Directorate has introduced focused antenatal care in all facilities which has enabled pregnant women to get direct health care from a particular health staff in the entire state of pregnancy.* (District Public Health Nurse)

The study found out that, in Ghana, the free maternal health-care services are available to women who are registered under the NHIS. This situation tends to prevent the less-privileged mothers to utilise services rendered by public health facilities because of the compulsory registration of pregnant women. Again, additional costs are attached to some services like screening tests. Such services offered to women are not covered by the free maternal health care under the NHIS. All respondents involved in the focus groups agreed that delivering in health facilities is the safest of all options. This was due to better management of pregnancy complications by health authorities. Notwithstanding, all respondents in the focus groups were deterred to utilise ANC in facilities with long waiting time, geographical constraints, high cost of additional charges, and poor attitude of caregivers. Some mothers in the focus groups also shared their experiences that other factors such as age of the mother, her education level, and the number of dependents, though personal, affected their pursuit of using antenatal care regardless of the free maternal care policy. A couple of expectant mothers from the focus groups shared their stories to the research team.

*This health insurance thing; you see it is a problem, you see me like this I do not work and my husband is a peasant farmer who cannot pay for this insurance thing. So the Member of Parliament (MP) in this area gathered people like me in this town to pay for us. You see now? So can I pay these additional costs all the time? It is a problem ooo….* (Pregnant woman, FG2; Sakora Wonoo)

### 4.3. Barriers to ANC Use

On other breadth, women recounted their desire to prefer the traditional birth attendant to the health facility due to the experiences their colleagues went through.

*I have three children already and this is the first pregnancy I'm attending the hospital. Hmm…this is why people like me do not come here ooo. The demands here are too much.* (Pregnant woman, FG2; Antoa)

This situation had the effect of either preventing women from utilising ANC at all or reducing their rate of attendance. The realities on the ground indicate that some women even visited facilities very late in their pregnancy so that they only needed to attend just once to check whether they are free from complications. The study showed that enabling factors like insurance and income outlined by Andersen [40] continue to determine the rate of health-seeking behaviour of women in Ghana even though maternal health services are supposed to be free. This is therefore likely to reduce the WHO recommendation of 4 antenatal visits before delivery [4, 34].

Geographical proximity, education, and long waiting time deter some pregnant women from using the ANC services. A critical situation of health need arises when long distance with high cost is incurred by expectant mothers to utilise health care. Though in emergency situations, long distances do not act as barriers to health-care utilisation. Such situations require quick responses to saving and sustaining lives. In analysing distance to health-care utilisation, Buor [41], in his study on the primacy of distance in the utilisation of health services in the Ahafo-Ano south district of Ghana, affirms that a patient with high income who does not see the need for health care would not access it even if the health facility is located closer to her compound, as someone who values the need for it due to high level of education would take risk of a loan to access health care even if the distance and service cost are so expensive. Geographical proximity constraints the provision of ANC services both health directorate in monitoring and supervision.

*Sir, me for instance, I travel for long time before I reach here and they also make me wait till I begin to talk and they say I like talking too much… errr, this is why I do not feel to come here sometimes ooo. The Nurses even say I do not have manners.* (Pregnant woman, FG1; Mamponteng)

*As I said earlier, we monitor and supervise the various facilities every fortnight and geographical constraint is one of the problems we do face as a directorate. We do get similar complaints from our clients due to the scattered settlement patterns of communities.* (District Public Health Nurse)

The study is therefore in consistent with the work of Buor [42], in his study on determinants of utilisation of health services by women in rural and urban areas in Ghana. Buor [42] discloses that restrictive factors as travel time, waiting time, quality of road, distance, transport cost, service cost, quality of service, and attitude of hospital staff deter clients from accessing health-care services. These conditions have direct or indirect influences on maternal access to health care, and they are more pronounced in the developing world including Ghana. Pregnant women are also constrained in using antenatal services in facilities that require long distances. A mother also shared her ordeal just before labour.

*If you arrive late to deliver, the midwives are harsh on you and start to rebuke you not considering the distance one has to bear before getting here.* (Recently delivered woman, FG1; Mamponteng)

The results revealed that women with high levels of education maximised their use of ANC compared to their counterparts without education. Hence, to improve maternal health-care services including ANC and to reduce maternal mortality in Ghana, there is the need to encourage and strengthen women's education to higher levels. The research observed that the knowledge of women on antenatal care was low on health education compared to the media and other educational platforms. Hence, it was observed that most expectant mothers were not well informed on the adequate level of ANC utilisation recommended by the WHO. There is therefore the need for state institutions such as the National Commission for Civic Education (NCCE) and the Information Services Department (ISD) to step up the education on maternal health-care use by using various community announcement channels. It is therefore imperative to perceive pregnancy as a journey between life and death; hence, expectant mothers must endeavour to utilise the recommended visits outlined by the WHO to avert complications and their associated mortalities. In Ghana, the causes of maternal mortality have been attributed to unaffordable health-care systems and inaccessible road networks [43–46].

Long waiting time was acknowledged by the study to influence the rate of ANC use. Access to maternal health care is a core pillar in public health and a matter of concern to all stakeholders for development [47]. Guagliardo [48] describes the actual time taken to utilise health care as “realised accessibility.” It is widely acknowledged that undue long waiting time on antenatal care at facilities reduces the utilisation of desired services rendered to patients [34, 49]. Particularly, women from the informal sector experience loss of productive hours waiting to access health care; hence, national productivity is reduced. Though most participants spent 20–40 minutes to access ANC in most of the facilities, in a particular facility, a respondent who always spent 40–60 minutes anytime she sought for health care shared her experience.

*Sir, the nurses in this facility are sometimes late for ANC sessions and even when they settle, they spend much time on us. So anytime I come for ANC, I do not go to the market to sell but my daughter sells for me instead.* (Recently delivered woman, FG1)

*I sometimes feel shy to come to see the Doctor here. Errr the last time I was here, erhm, the nurses gave me a long note of things I may need and you know I could not pronounce them and one of the Nurses scolded me all because I could not read the things to buy in the list. Oh! A small nurse ooo hmmm.* (Woman in third trimester, FG2)

This call is necessitated in all societies due to the productive ventures women get involved with and the intrinsic value of women's health, especially in developing countries like Ghana [50]. These delays during ANC sessions have called for the introduction of the focused ANC in some facilities. This is a policy which seeks to pair one pregnant woman to a health worker, preferably a nurse on a one-on-one basis who may be keen to know the overall history of the patient. Hence, expectant mothers are given spontaneous service in order to leave early for other things.

The observations of this study is in agreement with the research conducted by Chaibva [51] on factors influencing adolescents' utilisation of antenatal care services in Bulawayo, Zimbabwe, that pregnant adolescents were not satisfied with services rendered because of long delays in accessing ANC. There is therefore the need to achieve the Sustainable Development Goals 3 and 5 which seek to ensure healthy lives and promote well-being for all at all ages and empower women of all ages of which antenatal care is inclusive.

### 4.4. Attitude of Caregivers

Attitude of caregivers on ANC use presents mixed evidence on maternal health care in the Kwabre East district. In some facilities, attitude of caregivers was perceived to have stimulated the rate of ANC utilisation. Despite the experience of multiple child birth by some old women, they were still encouraged not to miss an ANC session schedule with health staff. Caregivers were seen to be open to all clients and operated their daily tasks in a friendlier environment. Hence, expectant mothers felt at home anytime there was the need to go for ANC visits. Findings from the study facilities highlight sensitive, caring, and more concerned to the plight and conditions of mothers. This was recounted by a nursing mother from a study facility in Sakra Wonoo.

*The nurses and midwives here take much care of patients. Sometimes when you feel dizzy, they get you a place to relax as if you are at home. They respect and smile to us anytime we go to receive care and are not bothered even if you go there late.* (Recently delivered woman, FG1)

Notwithstanding the above, attitude of caregivers was perceived as intimidating, rude, abusive, neglectful, and unfriendly by respondents in other facilities. These were reported as among the key reasons that either delayed or limited women from accessing antenatal care. It was revealed by mothers that the attitude of staff became worse when a mother was not registered under the NHIS and if she is about laboring or having complications.

*Sir, here is different ooo… they (health staff) do not care for us at all, when you go in the morning they will say why are you coming so early? When you go in the afternoon they will say why did not you come in the morning? When you go in the evening they will say they have closed, come the next day. In fact, they insensitive and do not respect us all.* (Pregnant woman, FG2)

In some situations, women reported being humiliated in inhumane manner though no fault of theirs.

*Sometimes, you do not feel like telling the Nurse what is wrong with you… when you see her scream at a sick pregnant woman.* (Woman in ninth month of pregnancy, FG2)

The findings therefore support the studies of Titaley et al. [52], Agboolah [53], Choudhury and Ahmed [54], and Akowuah et al. [4] that antenatal care attendance is responsive to the attitude of health staff. This phenomenon is able to impact either positively or negatively to effectively increase or decrease ANC turnout to the standard of the WHO recommendations. In effect, maternal mortality could be brought to the barest minimum if not eradicated at all in a more comprehensive maternal care.

## 5. Conclusion

This paper explored the conditions surrounding the use of (ANC), a major component of maternal health care in the Kwabre East district of Ghana using a set of interview and focus group discussions. The study sought to investigate the common realities on the ground on ANC use after the introduction of the free maternal health-care policy in tandem with the (WHO) recommendation of four visits before delivery. The findings disclose that the health directorate provides a wide range of maternal health-care services including distribution of health staff, routine monitoring of facilities, screening, immunisation, health education, and management of minor ailment. Using thematic analysis on four health facilities, the study reveals that education, waiting time, geographical constraints, additional charges to ANC, and attitude of caregivers still influence antenatal care use in the Kwabre East district. Even though the free maternal policy has increased antenatal care use in the district, adequate utilisation has not yet been achieved wholly because of the acknowledged factors.

The study recommends that to ensure adequate ANC utilisation in the Kwabre East district, the health directorate should offer support to the less-privileged mothers. This can be in the form of the absorption of screening costs and provision of drugs that are not covered by the policy, and also drugs should be easily available at facilities to mothers even if they are to be purchased. It is recommended that the focused antenatal care adopted by the health directorate should be scaled up nationally and extended to all facilities and supervised regularly. Antenatal care services should be reoriented and other policies from the directorate should take the upstream approach to ANC since it focuses on the determinants of maternal health. Also, women should be encouraged to pursue higher levels of education at least at the high school level. Health staff should be in-service trained on their attitude since some mothers, especially those without health insurance, felt intimidated because of possible abuse. To promote broader health systems, the government and the Kwabre East health directorate should collaborate with some state institutions whose primary purpose is to improve health such as the Information Services Department (ISD) and the National Commission for Civic Education (NCCE) to intensify campaigns on maternal health-care use. Community announcement systems should be incorporated in isolated areas where there is no or limited coverage of the mass media. Since these are preventive health-care measures, they can help reduce maternal mortality and assist Ghana attain the Sustainable Development Goal (Goals 3 and 5).

## Figures and Tables

**Table 1:** Global theme: pathways to evidence-based antenatal care.

Codes	Basic themes	Organising themes
Distribution of health staff	Improved health services	Improved ANC services
Routine monitoring of facilities	Improved ANC awareness	
Health supervision		
Health education and other services	Empowerment of mothers	

Protocol to work with	Increased ANC turnout	Focused ANC
Client-friendly antenatal care		
Daily ANC	Improved ANC reliance	
Intensive ANC		
Confidence and trust		
Relief for mothers		
Privacy of service rendered		

Geographical constraints	Inadequate logistics	ANC services are constrained
Delays in paying claims		
Financial barriers		
Inadequate nurses and doctors	Inadequate health staff	
Religious beliefs of clients		
Misconception on free health care	Free care is strictly by insurance	

Poor attitude of some caregivers	Attitude is deterrent to ANC	Attitude of health staff is vital
Insensitive of health staff		
Lack of respect for mothers		
Respect for mothers	Attitude of health staff	
Lateness of health staff		

Scientific proof	Rich sources of information	Evidence is crucial
Credibility of indication		
Evidence-based planning	Evidence is integrated	
Something proven to work		
Standardised protocol and procedures	Things we hear and testify	
Trust in evidence		

Source: the authors' construct (2016).
